# Effects of high-intensity interval training versus moderate-intensity continuous training on cardiorespiratory function in patients after stroke: a systematic review and meta-analysis of randomized trials

**DOI:** 10.3389/fneur.2026.1727980

**Published:** 2026-02-12

**Authors:** Ho-Wei Lin, Yuan-Chen Chang, Ting-Hsuan Hsu, Yen-Nung Lin

**Affiliations:** 1 Department of General Medicine, Wan Fang Hospital, Taipei Medical University, Taipei, Taiwan; 2 Department of Physical Medicine and Rehabilitation, Wan Fang Hospital, Taipei Medical University, Taipei, Taiwan; 3 Graduate Institute of Injury Prevention and Control, Taipei Medical University, Taipei, Taiwan

**Keywords:** endurance training, exercise, high-intensity interval training, meta-analysis, stroke

## Abstract

**Objective:**

Whether high-intensity interval training (HIIT) is more effective than moderate-intensity continuous training (MICT) in improving cardiorespiratory fitness (CRF) among patients after stroke remains unclear. We conducted this systematic review and meta-analysis to investigate the effects of HIIT versus MICT on CRF.

**Methods:**

We performed a literature search in the PubMed, Embase, and Cochrane Library from their earliest publication record to February 2025. Randomized trials comparing the outcomes of HIIT and MICT in patients after stroke were included. The mean difference (MD) and standardized mean difference (SMD) were determined by pooling the means and standard deviations of pretreatment–posttreatment changes for the CRF outcomes [i.e., oxygen consumption at peak (V̇O_2-peak_) and at ventilation threshold (VO_2-VT_)], mobility outcomes (i.e., walk endurance, speed, and postural balance) and training fidelity parameters (i.e., peak and mean heart rate during training sessions).

**Results:**

Nine articles, encompassing eight trials and a total of 371 patients, were included in the analysis. The meta-analysis revealed that HIIT was more effective in improving V̇O_2-peak_ (MD = 1.88 mL/kg/min, 95% CI: 1.20 to 2.55, *p* < 0.05) and VO_2-VT_ (MD = 2.20 mL/kg/min, 95% CI: 0.46 to 3.95, *p* < 0.05). However, HIIT did not show greater effectiveness in improving the 6-min walk test, 10-meter gait speed, or Berg Balance Score. Regarding training fidelity, a significantly higher mean heart rate [measured as a percentage of heart rate reserve (HRR, %)] was observed in HIIT sessions (MD = 19.36% HRR, 95% CI: 13.83 to 24.90, *p* < 0.05).

**Conclusion:**

HIIT is more effective than MICT in improving V̇O_2-peak_ and VO_2-VT_ in patients after stroke, supporting HIIT may serve as an alternative for aerobic training in this population.

**Systematic review registration:**

https://www.crd.york.ac.uk/PROSPERO/view/CRD42025645342, CRD42025645342.

## 1 Introduction

Stroke is the leading cause of disability and the third most common cause of death globally, affecting approximately 93.8 million people worldwide, and causing about 160.5 million disability-adjusted life-years lost in 2021 (1). Patients after chronic stroke often face motor impairments, leading to mobility challenges such as walking and balance difficulties (1). Reduced physical activity can further impact cardiorespiratory fitness (CRF), which is defined as the capacity of the circulatory and respiratory systems to supply oxygen to skeletal muscle mitochondria for energy production required during physical activity (2). Patients after stroke typically demonstrate low values of peak oxygen uptake (V̇O_2-peak_) (3), a key indicator of CRF. Therefore, improving CRF by implementing aerobic training in this population is critical in stroke rehabilitation (4).

Currently, moderate-intensity continuous training (MICT), typically targeting a heart rate of 40–80% of heart rate reserve (HRR), is the most commonly used aerobic training program and is recommended in stroke rehabilitation guidelines (5–7). The benefits of MICT in improving V̇O_2-peak_, motor function, and cardiovascular risk factors (e.g., blood pressure and glucose levels) are well established (8, 9). However, evidence suggests that the therapeutic effects of aerobic training may be associated with training intensity (10). The intensity of MICT may not sufficiently challenge the cardiovascular system to elicit maximal adaptations, nor meet the threshold required to complete many activities of daily living (10, 11).

Previous studies have proposed the use of high-intensity training (>60% of HRR) could augment outcomes, although such high intensity exercise can be challenging for patients after stroke (12, 13). Therefore, high-intensity interval training (HIIT), a modality that maximizes exercise intensity by alternating bursts of high intensity effort with recovery periods to enable higher sustained intensities at lower perceived exertion than high-intensity continuous exercise, has gained popularity in recent years (14–16).

Several meta-analyses have shown that HIIT leads to significantly better outcomes than MICT in healthy individuals and in patients with chronic diseases such as cancer, obesity, coronary artery disease, and heart failure (17–23). However, few clinical trials have compared the effects of HIIT and MICT in patients after stroke, leaving its therapeutic efficacy unclear. To date, only two meta-analyses have explored the effects of HIIT among patients after stroke. Both of them primarily compared HIIT with control group (usual care) or low-intensity continuous training, which lead to obviously better results (24, 25). With emerging clinical trials that explores the difference between HIIT and MICT in stroke population, we conducted this review study to provide further insight for clinical practice.

In the present study, we aimed to explore the effects of HIIT versus MICT on improving CRF, functional performance in mobility, and the differences in training fidelities between HIIT and MICT.

## 2 Methods

This systematic review and meta-analysis was registered in the International Prospective Register of Systematic Reviews of the UK National Institute for Health Research (PROSPERO; ID: CRD42025645342), and was performed in accordance with Preferred Reporting Items for Systematic Reviews and Meta-Analyses (PRISMA) guidelines (50).

### 2.1 Searching strategy

We searched the PubMed, Embase, and Cochrane Library for studies published from inception to February 2025 using the following search terms: ([high-intensity interval training OR HIIT] OR [moderate-intensity continuous training OR MICT] OR aerobic training) AND (stroke OR cerebrovascular accident OR cerebrovascular disorder OR cerebral infarction OR brain infarction OR intracranial arteriosclerosis OR intracranial thrombosis OR intracranial embolism OR CVA). The detailed searching strategy is presented in the Supplementary Data 1. Moreover, all retrieved abstracts, studies, and citations were reviewed. No language restrictions were applied.

### 2.2 Study inclusion and exclusion criteria

We included randomized trials on the basis of their titles and abstracts in accordance with the following selection criteria: (1) compared the outcomes of HIIT with MICT in patients after stroke, and (2) reported the inclusion and exclusion criteria for patient selection. We excluded trials that (1) were observational or nonrandomized trials, (2) primarily used intervention other than HIIT (ie, high intensity continuous training), (3) compared HIIT with non-MICT intervention (ie, low intensity exercise or usual care), and (4) had not been published as full-text articles in a peer-reviewed journal.

### 2.3 Data extraction

Two reviewers independently extracted baseline and outcome data, including the number, age, and sex of the participants; inclusion and exclusion criteria; number of patients after ischemic stroke; intervention regimens, frequency, and duration; and outcome parameters. Individually recorded data from two reviewers were compared, and any disagreements were resolved by a third reviewer. In case of missing data or interesting data that were not reported in the article, the corresponding authors of the original study for information were contacted through e-mail.

### 2.4 Outcomes

The primary outcome was the CRF indicators, including V̇O_2-peak_ (mL/kg/min) or VO_2_ at ventilation threshold (VO_2-VT_, mL/kg/min) measured by cardiopulmonary exercise test. Previous studies have determined the minimum detectable change for V̇O_2-peak_ in patients after stroke to be 1 mL/kg/min (26, 27). The secondary outcomes were the functional performance in mobility, involving walking speed (10-meter walk speed), walking endurance (6-min walk test), and postural balance (Berg Balance Score). Resting blood pressure, a cardiovascular risk indicator, was surveyed. In addition, parameters of training fidelity representing the intensity the patient sustained during a training session were included.

### 2.5 Study quality assessment

The methodological quality of the randomized trials was assessed independently by two reviewers, in accordance with the revised Cochrane Risk of Bias (RoB 2.0) tool, PEDro scale, and the Tool for the Assessment of Study Quality and Reporting in Exercise (TESTEX).

RoB 2.0 includes the following domain: bias deriving from the randomization process, bias caused by deviations from intended interventions, bias caused by missing outcome data, bias arising from outcome measurement, bias deriving from selection of the reported results, and overall risk of bias (28). Each domain was rated as having low risk of bias, some concerns, or high risk of bias. The PEDro scale assesses the quality of randomized controlled trials in physiotherapy and rehabilitation, focusing on validity, statistics, and design. It includes 11 criteria, with scores below 4 rated as poor, 4–5 as fair, 6–8 as good, and 9–10 as excellent (29). The TESTEX scale is a 15-point scale that is designed specifically for use in exercise training studies, with scores below 6 rated as ‘low quality’, 7–11 as ‘good quality’, and 12–15 as ‘high quality’ (30). After individual assessments, the two reviewers discussed any potential discrepancies, which were subsequently resolved by a third reviewer.

### 2.6 Data synthesis and presentation

Data were analyzed by Review Manager, Version 5.3 (Cochrane Collaboration, Oxford, England). Standard deviations were estimated from the provided confidence interval (CI) limits or standard errors. For the primary and secondary outcomes, the means and standard deviations of pretreatment–posttreatment changes were used in the meta-analysis. When necessary, these values were estimated in accordance with the reported pretreatment and posttreatment data (31). Continuous outcomes were analyzed using the mean difference (MD) or the standard mean difference (SMD). The precision of the effect sizes was reported as 95% CIs. We used the DerSimonian and Laird random-effects model to compute a pooled estimate of the MD or the SMD (32). The inverse variance method was used to analyze continuous variables. The *I*^2^ test was used to quantify the heterogeneity of the outcomes. Heterogeneity was classified as small, moderate, and large for *I*^2^ values of 25, 50, and 75%, respectively. Results were reported only when 2 or more studies were available for meta-analysis on the same outcome.

### 2.7 Assessment of quality of evidence

We used the Grading of Recommendation Assessment, Development, and Evaluation (GRADE) approach to access the quality of evidence (33). Rating aspects include (1) study limitations, (2) inconsistency, (3) indirectness, (4) imprecision, and (5) publication bias. We consequently graded the quality of evidence for each outcome as high, moderate, low, or very low quality.

## 3 Results

### 3.1 Search results and study characteristics

**Figure 1:**
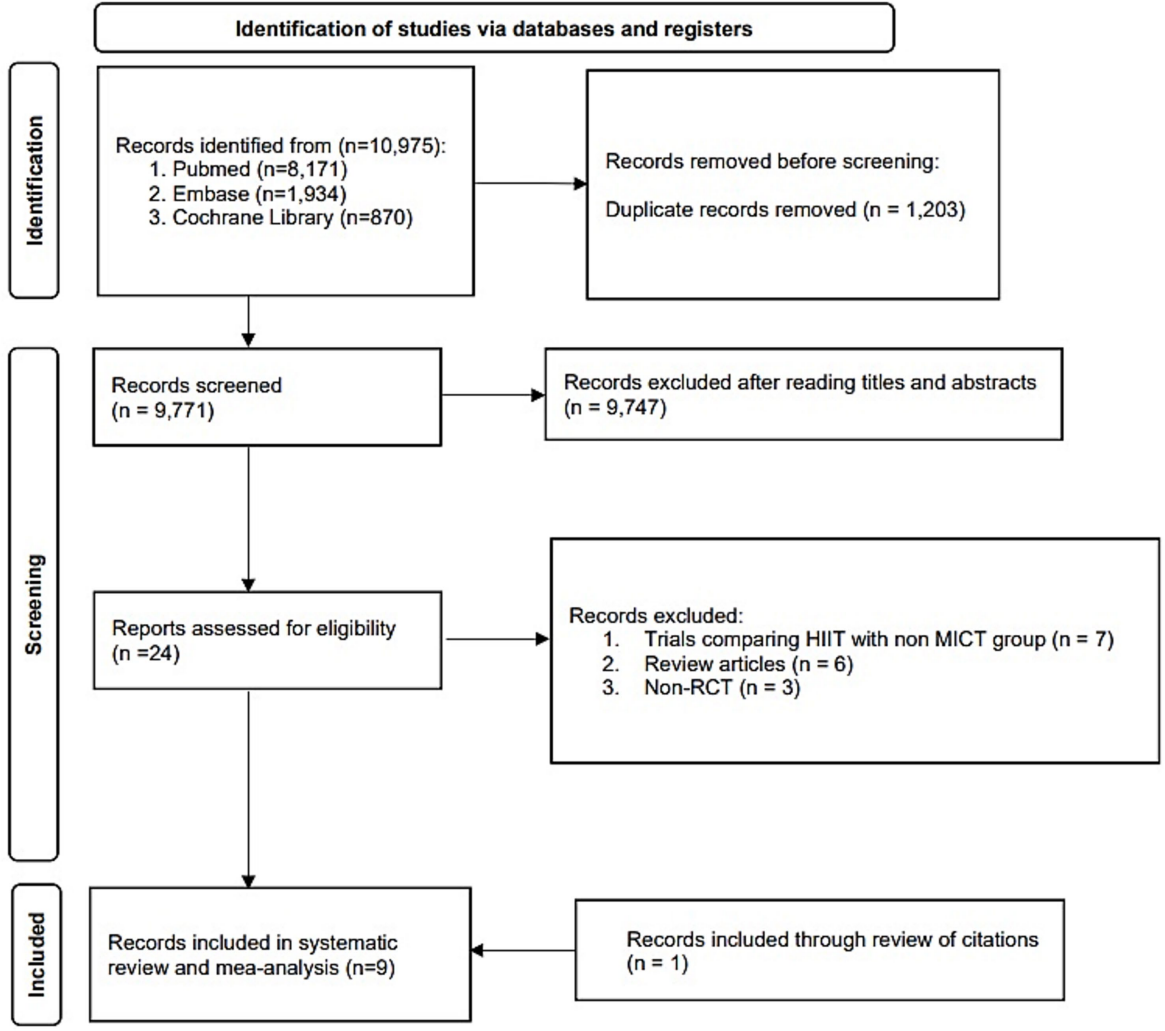
Flowchart of study identification and selection.

A flowchart describing the screening and selection process is presented in Figure 1. Nine articles involving eight different trials were included in the systematic review and meta-analysis (34–42).

The characteristics of the included trials are summarized in Table 1. Two articles reported different aspects of the same trial; one concerns CRF benefits (40) and the other concerns psychosocial responses (41). Three trials were conducted in Canada (37, 39–41), two in The United States of America (34, 35), one in Pakistan (38), one in South Korea (42), and one in Taiwan (36). Two articles reported different aspects of the same trial; one concerns CRF benefits and the other concerns psychosocial responses after training (40, 41). A total of 371 patients aged from 50 to 80 were included in the meta-analysis.

**Table 1:** Characteristics of included studies.

Author year; country	Selection criteria	Number of patients (% male)	Age, year, mean ± SD	Number of patients with ischemic stroke (%)	Intervention					PEDro scale
					Modality	Intensity	Duration (min/sess)	Frequency (sess/wk)	Intervention period (wks)	
Boyne et al. (2016) (35); USA	Ambulatory, poststroke duration > 6 months	H: 11 (63.6) M: 5 (40)	H: 59 ± 9 M: 57 ± 12	H: 9 (81.8) M: 2 (40)	H: Treadmill exercise M: Treadmill exercise	H: 30-s bursts of HI walking, alternated with 30- to 60-s LI periods (treadmill stopped). Average session HR progressed from a mean 53% HRR in 1st week to 72% HRR in 4th week. M: Walking with speed adjusted to maintain 45% HRR, then progressed to 50% HRR after 2 wks of training.	H: 25 M: 25	H: 3 M: 3	H: 4 M: 4	8/10
Boyne et al. (2023) (34); USA	Patients after a single stroke	H: 27 (59.3) M: 28 (71.4)	H: 63.8 ± 9.9 M: 61.5 ± 9.9	H: 15 (55.6) M: 19 (67.9)	H: Walking practice and treadmill exercise M: Walking practice and treadmill exercise	H: 30-s bursts of HI walking, alternated with 30- to 60-s LI periods; targeting a mean aerobic intensity above 60% of the HRR. M: walking with speed adjusted to maintain a target HR of 40% of the HRR, progressing by 5% of the HRR every 2 weeks up to 60% of the HRR.	H: 45 M: 45	H: 3 M: 3	H:12 M: 12	8/10
Hsu et al. (2021) (36); Taiwan	Poststroke duration > 3 months	H: 10 (80) M: 13 (92.3)	H: 58.5 ± 8.7 M: 53.1 ± 6.9	H: 6 (60) M: 9 (69.2)	H: Bicycle ergometer M: Bicycle ergometer	H: Five 3-min intervals at 80% VO_2_ peak, each interval separated by 3 min of exercise at 40% of VO_2_ peak, for 30 min M: 60% VO_2_ peak for 30 min	H: 30 M: 30	H: 2–3 M: 2–3	Totally 36 sessions	6/10
Lapointe et al. (2023) (37); Canada	Ischemic stroke, poststroke duration > 3 months	H: 19 (68.4) M: 16 (62.5) C: 17 (58.8)	H: 71.8 ± 9.9 M: 65.6 ± 11.3\u00B0C: 69.6 ± 10.7	H: 19 (100) M: 16 (100) C: 17 (100)	H: Ergocycle training + home exercise. M: Ergocycle training + home exercise. C: Usual care without any additional physical activity.	H: Bouts at 95% of peak power output interspersed with a 60-s recovery M: 50% of peak power output (Supervised training)	H: 20–40 M: 20–40	H: 3 sess/wk. of HIIT for 2 months, then 2 sess/wk. of HIIT + 1 sess/wk. of unsupervised MICT for the next 2 months, and then 1 sess/wk. of HIIT + 2 sess/wk. of unsupervised MICT for the next 2 months. M: 1 sess/wk. supervised MICT + 2 sess/wk. of unsupervised MICT for 6 months.		4/10
Mahrukh et al. (2023) (38); Pakistan	Poststroke duration > 6 months	H: 30 (NI) M: 30 (NI)	Patients aged 50–80 years old were included.	NI	NI	NI	NI	NI	H: 12 M: 12	4/10
Marzolini et al. (2023) (39); Canada	Poststroke duration > 10 weeks	H: 24 (83.3) M: 23 (78.3)	H: 62.8 ± 13.2 M: 60.9 ± 8.4	H: 16 (66.7) M: 20 (87.0)	H: Treadmill exercise M: Treadmill exercise	H: Alternative HI and LI intervals. HI: RPE of ≥17, LI: RPE of 10–12. M: HR_-VT_, RPE of 12–16, or 60–80% of HRR	H: 20–22 M: ≤60	H: 3 HIIT + 2 MICT M: 5 MICT	H: 24 M: 24	8/10
Moncion et al. (2024) (25) and Rodrigues et al. (41); Canada	Poststroke duration between 6 to 60 months	H: 42 (64.3) M: 40 (57.5)	H: 65.4 ± 8.9 M: 64.4 ± 9.7	H: 30 (71.4) M: 33 (82.5)	H: Adaptive recumbent steppers M: Adaptive recumbent steppers	H: 10 × 1-min bouts of HI exercise, interspersed with 9 × 1-min LI intervals, for 19 min. HI intervals targeted 80% HRR and progressed by 10% every 4 weeks up to 100% HRR. LI intervals targeted 30% HRR. M: Targeted 40% HRR for 20 min and progressed by 10% HRR and 5 min every 4 weeks, up to 60% HRR for 30 min	H: 19 M: 20–30	H: 3 M: 3	H: 12 M: 12	6/10
Soh et al. (2020) (42); South Korea	Patients after minor stroke	H: 18 (72.2) M: 18 (66.7)	H: 56.3 ± 5.3 M: 57.4 ± 7.2	H: 13 (72.2) M: 12 (66.7)	H: Lateral push-off skater exercise M: Conventional treadmill aerobic exercise	H: NI for intensity regarding HI and LI intervals. RPE ≤ 14 for a HIIT session was reported. M: RPE ≤ 14 and 40–80% of HRR	H: 30 M: 30	H: 3 M: 3	H: 12 M: 12	5/10

C, Control group; H, HIIT group; HI, high-intensity; HRR, Heart rate reserve; HR_-VT_, Heart rate at ventilation threshold measured by cardiopulmonary exercise test. M, MICT group; LI, low-intensity; NI, No information; RPE, rating on perceived exertion; sess, sessions.

In the training modality, three trials used treadmill exercise as their HIIT program (34, 35, 39), two trials used bicycle ergometer (36, 37), one trial used adaptive recumbent steppers (40, 41), one trial used lateral push-off skater exercise (42), and the other trial did not provide information regarding training modality (38). The intensity of high-intensity intervals was measured by heart rate (60–100% of HRR) in three trials (34, 35, 40, 41), VO_2_ (80–100% of V̇O_2-peak_) in two trials (36, 39), and power output in one trial (37). Two trials did not provide detailed information on defining the intensity of interventions (38, 42), though we have contacted the corresponding authors for more detailed information. Training duration and frequency varied from two to five sessions per week (34–37, 39–42); and the duration of intervention lasted for 4 weeks in one trial (35), 12 weeks in five trials (34, 36, 38, 40–42), and 24 weeks in the other two trials (37, 39).

### 3.2 Methodological quality

The assessment of ROB 2.0 of the included trials is summarized in Table 2 and Supplementary Figure 1. Overall, one trial was rated as having high risk of bias (38), one trial was rated as having moderate risk of bias (37), and the remaining six trials were rated as having low risk of bias (34–36, 39–42).

**Table 2:** Methodological quality assessment of the selected randomized controlled trials (RoB 2.0).

Study	Bias from the randomization process	Bias caused by deviations from intended interventions	Bias caused by missing outcome data	Bias in measurement of the outcome	Bias in selection of the reported results	Overall risk of bias
Boyne et al. (35)	Low risk	Low risk	Low risk	Low risk	Low risk	Low risk
Boyne et al. (34)	Low risk	Low risk	Low risk	Low risk	Low risk	Low risk
Hsu et al. (36)	Low risk	Low risk	Low risk	Low risk	Low risk	Low risk
Lapointe et al. (37)	Low risk	Low risk	Low risk	Moderate risk^a^	Low risk	Moderate risk
Mahrukh et al. (38)	Moderate risk^b^	Moderate risk^c^	Low risk	Moderate risk^d^	Low risk	High risk
Marzolini, et al. (39)	Low risk	Low risk	Low risk	Low risk	Low risk	Low risk
Moncion et al. (25) and Rodrigues et al. (41)	Low risk	Low risk	Low risk	Low risk	Low risk	Low risk
Soh et al. (42)	Low risk	Low risk	Low risk	Low risk	Low risk	Low risk

^a^No blinded process was conducted in the study. ^b^No detailed information regarding randomization process was provided. ^c^No detailed information regarding intervention regimen was provided. ^d^Whether blinded process was conducted was not mentioned.

The assessment of PEDro scale of the included trials is summarized in Table 1 and Supplementary Table 1. The PEDro scores ranged from four to eight. Most of the trials were downgraded due to lack of subject blinding and therapist blinding. Overall, three trials were rated as having fair methodological qualities (37, 38, 42), and the remaining six trials were rated as having good methodological qualities (34–36, 39–41).

The assessment of the TESTEX scale of the included trials is summarized in Supplementary Table 2. The TESTEX scores ranged from 10 to 15. Overall, two trials were rated as having good methodological qualities (37, 38), and the remaining six trials were rated as having high methodological qualities (34–36, 39–42).

The assessment results of the three assessment tools are generally consistent with each other.

### 3.3 Effects on CRF outcomes

**Figure 2:**
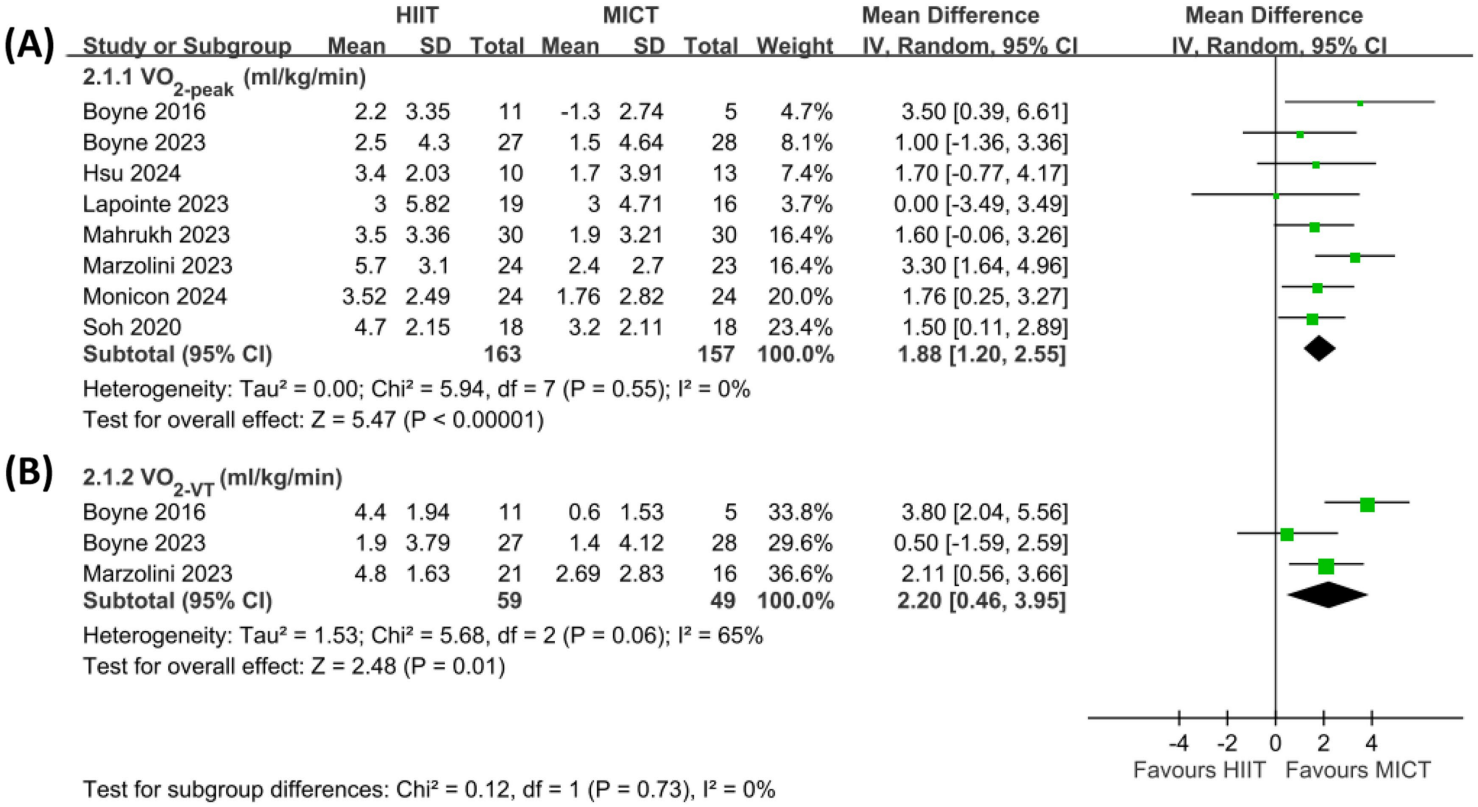
Forest plot presenting a comparison of changes in **(A)** VO_2-peak_ and **(B)** VO_2-VT._

Effects on V̇O_2-peak_ and VO_2-VT_ were shown in Figure 2. The pooled results from all included studies (34–42) showed that HIIT exhibited a significantly greater improvement in V̇O_2-peak_ than MICT (MD = 1.88 mL/kg/min, 95% CI = 1.20 to 2.55, *p* < 0.00001; Figure 2A). A sensitivity analysis excluding 2 studies (37, 38) with moderate and high risk showed a similar result (MD = 2.02 mL/kg/min, 95% CI = 1.27 to 2.77, *p* < 0.00001). In addition, the pooled results from three included studies (34, 35, 39) showed that HIIT exhibited a significantly greater improvement in VO_2-VT_ than MICT (MD = 2.20 mL/kg/min, 95% CI = 0.46 to 3.95, *p* = 0.01; Figure 2B).

### 3.4 Effects on functional performance in mobility

**Figure 3:**
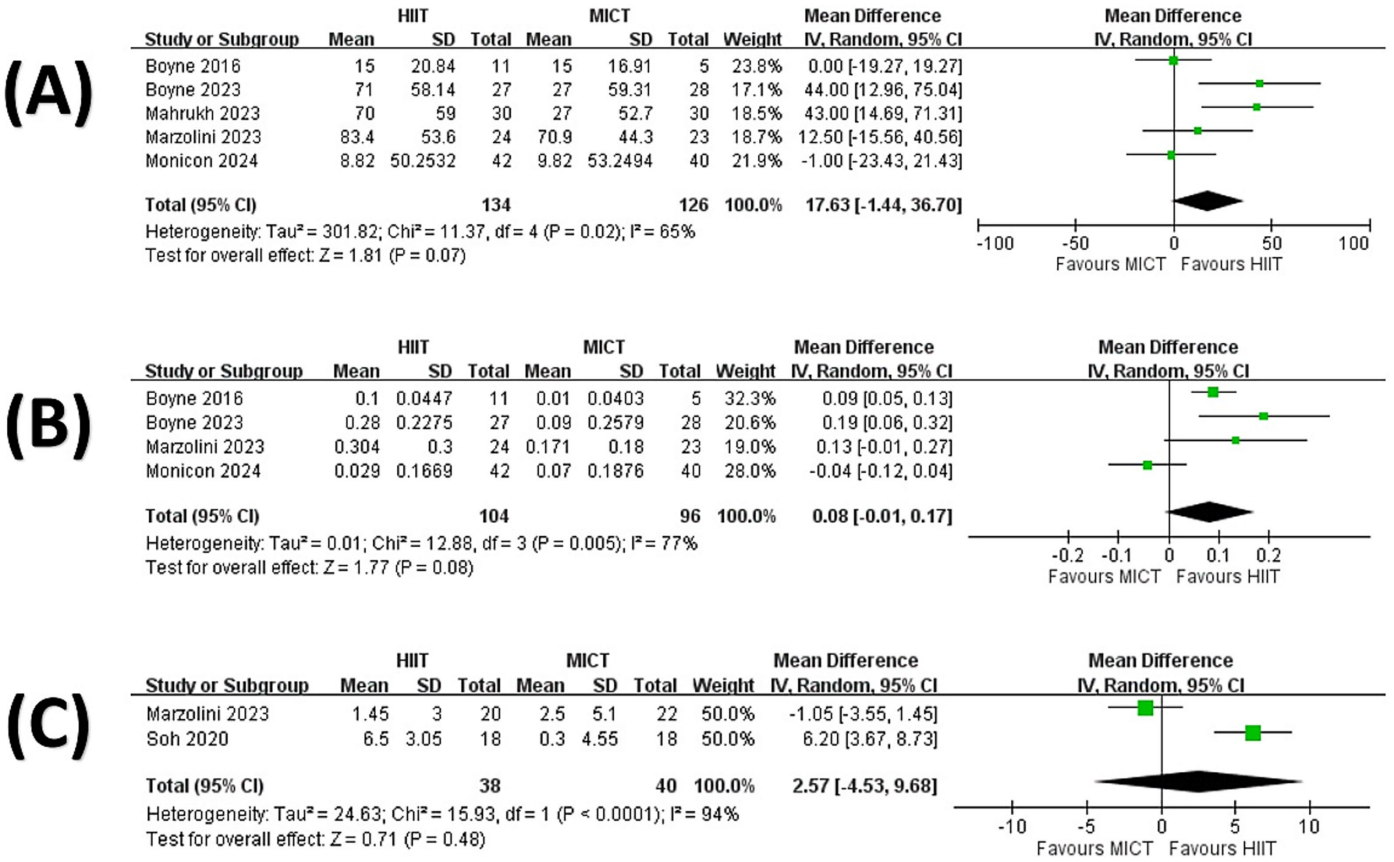
Forest plot presenting a comparison of changes in motor function: **(A)** 6-min walk test; **(B)** 10-meter gait speed; **(C)** Berg Balance Score.

The pooled results revealed that there was no significant difference between HIIT and MICT in change in 6-min walk test (MD = 17.63 m, 95% CI = −1.44 to 36.70, *p* = 0.07; Figure 3A) (34, 35, 38–40), the change in 10-meter gait speed (MD = 0.08 m/s, 95% CI = −0.01 to 0.17, *p* = 0.08; Figure 3B) (34, 35, 39, 40), or the change in Berg Balance Score (MD = 2.57, 95% CI = −4.53 to 9.68, *p* = 0.48; Figure 3C) (39, 42).

### 3.5 Training fidelity regarding the intensity

**Figure 4:**
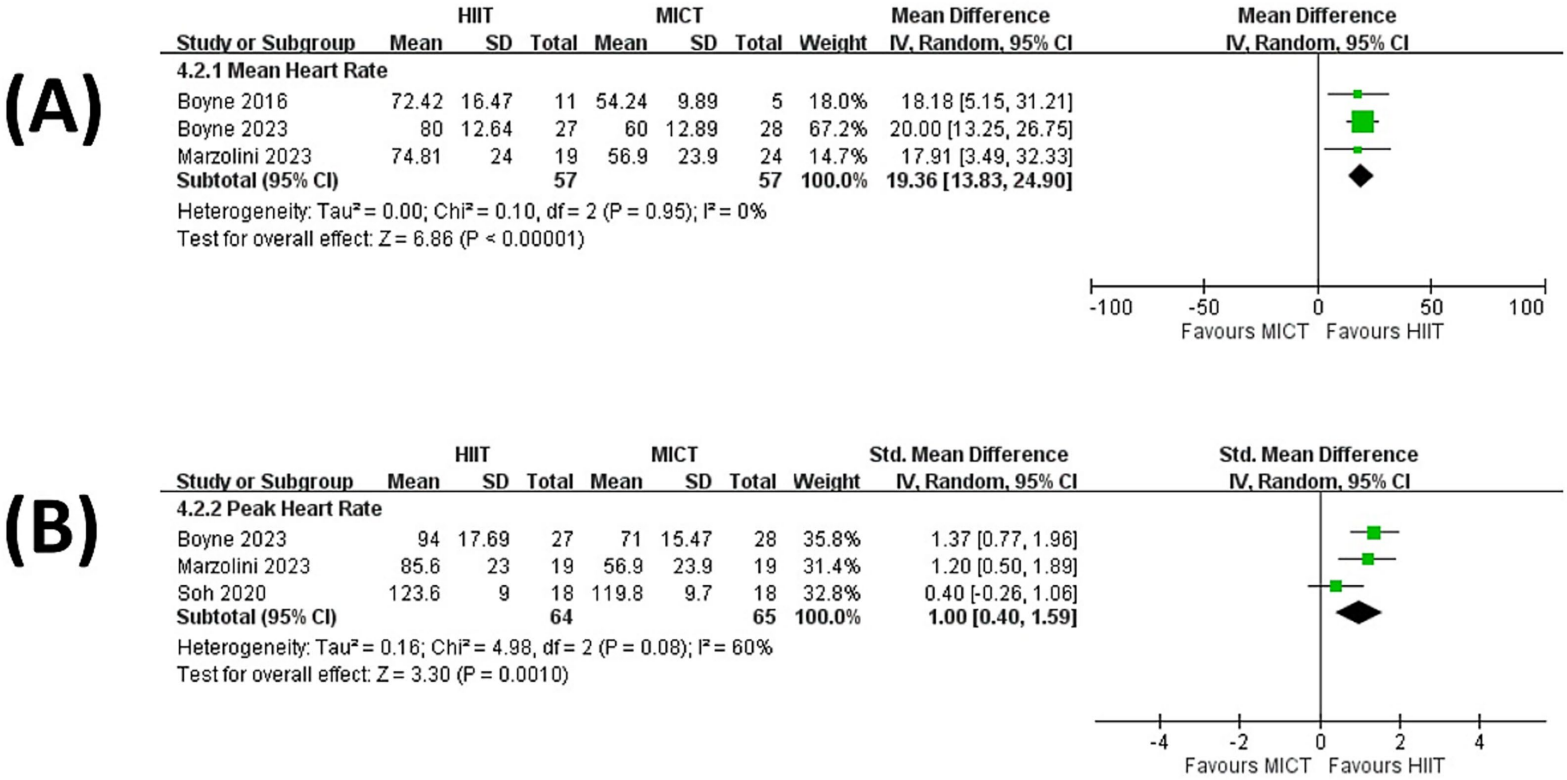
Forest plot presenting a comparison of changes in training fidelity: **(A)** Mean heart rate and **(B)** Peak heart rate.

The pooled results from three studies (34, 35, 39) showed that patients undergoing HIIT sustained greater mean heart rate than MICT during the training session (MD = 19.36% of HRR, 95% CI = 13.83 to 24.90, *p* < 0.00001; Figure 4A). In addition, patients undergoing HIIT also sustained greater peak heart rate than MICT during the training session (SMD = 1.00, 95% CI = 0.40 to 1.59, *p* = 0.0010; Figure 4B) (34, 39, 42).

### 3.6 Effects on resting blood pressure

During the intervention period, no significant differences were observed between HIIT and MICT in systolic blood pressure changes (MD = −1.72 mmHg, 95% CI = −5.92 to 2.48, *p* = 0.42; Supplementary Figure 2A) (37, 39, 40, 42) and diastolic blood pressure (MD = −1.04 mmHg, 95% CI = −3.78 to 1.69, *p* = 0.48; Supplementary Figure 2B) (37, 39, 40, 42).

### 3.7 Adverse effects

No adverse events of HIIT were observed in two of the included studies (37, 42). In another four studies, no study-related serious adverse events occurred, and the number of adverse effects was similar between the HIIT group and the MICT group. Common adverse events included pain, soreness, fatigue, lightheadedness, and fall (34, 35, 39, 40). No information on adverse effects was provided in the other two studies (36, 38).

### 3.8 Quality of evidence

The summary of findings and the grading of the evidence certainty are presented in Supplementary Table 3. The GRADE certainty of the evidence of the above outcomes were moderate or low because of inconsistency [unexplained heterogeneity was detected in the pooled result (*I*^2^ > 50%)] and imprecision (the number of studies and patients are small).

## 4 Discussion

To the best of our knowledge, this is the first systematic review to directly compare the effects of HIIT and MICT in patients after stroke. Overall, HIIT generated a 1.88 mL/kg/min improvement in V̇O_2-peak_ compared to MICT, which exceeded the reported minimum detectable change (26, 27) (1 mL/kg/min) in patients after stroke. Given that every 3.5 mL/kg/min (equal to 1 metabolic equivalent) increase in V̇O2-peak was associated with a 15 to 20% decrease in overall mortality among various populations (51), a difference of 1.88 mL/kg/min of V̇O_2-peak_ was clinically meaningful. Considering that MICT remained the most commonly used training mode in stroke rehabilitation, our results support the suggestion that HIIT may be also used as an alternative for aerobic training in stroke population.

Previous studies have shown that acute bout of HIIT activates signaling pathways involving mitochondrial biogenesis, and repeated activation of these pathways may lead to specific muscular adaptations, including increased mitochondrial density, capacity and respiration in skeletal muscle (43–45), which is related to improvements in V̇O2-peak. In addition, previous studies have shown that HIIT can lead to better improvements in cardiac contractility, systolic function, and endothelial function in patients with cardiovascular diseases when compared to MICT (21, 46). Moreover, one included study in our meta-analysis also compared the physiological effects of HIIT to those of MICT on patients after stroke, and the results showed that HIIT significantly improved cardiac output (mean improvement: 1.45 L/min, *p* = 0.038) and serum brain-derived neurotrophic factor level (mean improvement: 1.85 ng/mL, *p* = 0.012). Improvement in aerobic capacity by increasing systemic tissue oxygen extraction, and increased cerebral oxygen utilization in the involved hemisphere was also observed after HIIT when compared with MICT (36). All these mechanisms discussed above may lead to better improvements in CRF in the HIIT group when compared with the MICT group, which is the primary result in this meta-analysis.

However, our results revealed that HIIT did not result in superior functional mobility improvements, indicating that HIIT is not superior to MICT in those variables. This may be attributed to the relatively small number of included studies assessing these outcomes and the high heterogeneity observed (e.g., *I*^2^ = 65% for the 6-min walk test and 94% for the Berg Balance Score), suggesting variability in patient characteristics and intervention protocols. While CRF plays a crucial role in mobility, stroke-related impairments such as neuromuscular dysfunction, reduced motor coordination, muscle weakness, proprioceptive deficits, and impaired balance may limit the direct translation of improved aerobic capacity into functional mobility gains (47). Further research is needed to better understand the effects of HIIT on functional mobility in patients after stroke and to identify factors that may influence its efficacy.

Two recent review studies have addressed the effects of HIIT in the stroke population (24, 25). Although Anjos et al. supported the superiority of HIIT over continuous aerobic training, their conclusion was based on only 4 trials with 91 patients. Moreover, their continuous aerobic training group was mixed with low-intensity walking, which caused bias in interpreting the true difference between HIIT and MICT (24). On the other hand, Moncion et al. conducted a systematic review on the effects of various aerobic exercise interventions (i.e., HIIT, high-intensity continuous training, MICT, low-intensity continuous care, usual care) (25). Their network meta-analysis showed no significant mean difference between HIIT and MICT (i.e., MD = 0.82 mL/kg/min, 95%CI: −0.92 to 2.56). It should be noted that their result was based on only 4 trials involving direct comparison between HIIT and MICT. Additionally, the methodological bias arising from indirect comparison in network meta-analysis was also concerned (48). In contrast, we updated the knowledge with 4 new trials and provided more solid evidence regarding the comparison between HIIT and MICT.

Although our results supported the superiority of HIIT over MICT in improving V̇O2-peak, the underlying mechanism remained unclear. Our meta-analysis showed that both peak and mean heart rate was higher during HIIT sessions than during MICT sessions (Figure 4). This finding may imply that the HIIT session provided higher averaged training intensity than the MICT did. Since it has been well established that the effect of aerobic training on CRF is associated with training intensity (52), a higher averaged training intensity may contribute to the superiority of HIIT. This notion should be confirmed with more evidence involving the comparison regarding the training fidelity between HIIT and MICT.

Training intensity (i.e., high or moderate) and duration (i.e., interval or continuous or time spent on a session) are two controlling factors of training volume. It is rational to compare two interventions at different intensity but with same duration, or with different duration but at same intensity. However, comparing two training modes that differ in both intensity and duration leads to methodological challenges. At least one included study controlled for training volume and still found evidence favoring HIIT. In the study by Hsu et al., the HIIT protocol consisted of alternating intervals at 40 and 80% of V̇O_2-peak_, while the MICT protocol was performed at a steady intensity of 60% V̇O_2-peak_, with a matched total training duration (36). This isoenergetic expenditure design minimized the confounding effect of training volume. We advocate for future trials to control training volume (i.e., the integral of training intensity and duration) when comparing HIIT and MICT. Doing so will provide clearer insights into the underlying factors contributing to HIIT’s potential superiority.

## 5 Limitations

Our study has several limitations. First, the number of included randomized trials and the sample sizes were relatively small, potentially affecting result precision. Larger randomized trials should be conducted to compare the effects of HIIT and MICT in patients after stroke. Moreover, variations in cardiopulmonary exercise test protocols, exercise intervention regimens, frequency, and duration among trials may cause considerable heterogeneity. In addition, previous article has stated that V̇O_2-peak_ may not precisely represent CRF, especially in clinical population (49). However, only two CRF indicators (V̇O_2-peak_ and VO_2-VT_) were available for data pooling. Future studies may investigate the effects of HICT on different CRF indicators. Finally, only the changes between pretreatment and posttreatment data were analyzed; therefore, leaving the long-term sustainability of improvement unclear.

## 6 Conclusion

In conclusion, our results indicate that no difference was detected between HITT and MICT for functional mobility. However, HIIT significantly improves CRF indicators compared to MICT in patients after stroke (moderate certainty evidence on GRADE), supporting HIIT as a preferred option over MICT for enhancing cardiorespiratory fitness in this population.

## Data Availability

The original contributions presented in the study are included in the article/Supplementary material, further inquiries can be directed to the corresponding author/s.

## References

[1] GBD 2021 Stroke Risk Factor Collaborators. Global, regional, and national burden of stroke and its risk factors, 1990–2021: a systematic analysis for the global burden of disease study 2021. Lancet Neurol. (2024) 23:973–1003. doi: 10.1016/S1474-4422(24)00369-7, 39304265 PMC12254192

[2] Raghuveer G Hartz J Lubans DR Takken T Wiltz JL Mietus-Snyder M . Cardiorespiratory fitness in youth: an important marker of health: a scientific statement from the American Heart Association. Circulation. (2020) 142:e101–18. doi: 10.1161/cir.0000000000000866, 32686505 PMC7524041

[3] Billinger SA Coughenour E Mackay-Lyons MJ Ivey FM. Reduced cardiorespiratory fitness after stroke: biological consequences and exercise-induced adaptations. Stroke Res Treat. (2012) 2012:959120. doi: 10.1155/2012/959120, 21876848 PMC3159380

[4] Cheary S Levy T Ramos JS Lange B. Understanding how cardiorespiratory training is implemented to address cardiorespiratory fitness in adults following a stroke: a systematic review. Disabil Rehabil. (2025) 47:4297–315. doi: 10.1080/09638288.2024.2449397, 39804024

[5] Billinger SA Arena R Bernhardt J Eng JJ Franklin BA Johnson CM . Physical activity and exercise recommendations for stroke survivors: a statement for healthcare professionals from the American Heart Association/American Stroke Association. Stroke. (2014) 45:2532–53. doi: 10.1161/STR.0000000000000022, 24846875

[6] Mackay-Lyons M Billinger SA Eng JJ Dromerick A Giacomantonio N Hafer-Macko C . Aerobic exercise recommendations to optimize best practices in care after stroke: AEROBICS 2019 update. Phys Ther. (2020) 100:149–56. doi: 10.1093/ptj/pzz153, 31596465 PMC8204880

[7] Winstein CJ Stein J Arena R Bates B Cherney LR Cramer SC . Guidelines for adult stroke rehabilitation and recovery: a guideline for healthcare professionals from the American Heart Association/American Stroke Association. Stroke. (2016) 47:e98–e169. doi: 10.1161/STR.0000000000000098, 27145936

[8] Brouwer R Wondergem R Otten C Pisters MF. Effect of aerobic training on vascular and metabolic risk factors for recurrent stroke: a meta-analysis. Disabil Rehabil. (2021) 43:2084–91. doi: 10.1080/09638288.2019.1692251, 31794269

[9] Saunders DH Sanderson M Hayes S Johnson L Kramer S Carter DD . Physical fitness training for stroke patients. Cochrane Database Syst Rev. (2020) 3:Cd003316. doi: 10.1002/14651858.CD003316.pub6, 32196635 PMC7083515

[10] Boyne P Welge J Kissela B Dunning K. Factors influencing the efficacy of aerobic exercise for improving fitness and walking capacity after stroke: a Meta-analysis with Meta-regression. Arch Phys Med Rehabil. (2017) 98:581–95. doi: 10.1016/j.apmr.2016.08.484, 27744025 PMC5868957

[11] Marzolini S Wu CY Hussein R Xiong LY Kangatharan S Peni A . Associations between time after stroke and exercise training outcomes: a meta-regression analysis. J Am Heart Assoc. (2021) 10:e022588. doi: 10.1161/JAHA.121.022588, 34913357 PMC9075264

[12] Boyne P Dunning K Carl D Gerson M Khoury J Kissela B. High-intensity interval training in stroke rehabilitation. Top Stroke Rehabil. (2013) 20:317–30. doi: 10.1310/tsr2004-317, 23893831

[13] Reynolds H Steinfort S Tillyard J Ellis S Hayes A Hanson ED . Feasibility and adherence to moderate intensity cardiovascular fitness training following stroke: a pilot randomized controlled trial. BMC Neurol. (2021) 21:132. doi: 10.1186/s12883-021-02052-8, 33745454 PMC7983371

[14] Gibala MJ Little JP Macdonald MJ Hawley JA. Physiological adaptations to low-volume, high-intensity interval training in health and disease. J Physiol. (2012) 590:1077–84. doi: 10.1113/jphysiol.2011.224725, 22289907 PMC3381816

[15] Lock M Yousef I Mcfadden B Mansoor H Townsend N. Cardiorespiratory fitness and performance adaptations to high-intensity interval training: are there differences between men and women? A systematic review with Meta-analyses. Sports Med. (2024) 54:127–67. doi: 10.1007/s40279-023-01914-0, 37676620 PMC10799129

[16] Poon ET Li HY Little JP Wong SH Ho RS. Efficacy of interval training in improving body composition and adiposity in apparently healthy adults: an umbrella review with Meta-analysis. Sports Med. (2024) 54:2817–40. doi: 10.1007/s40279-024-02070-9, 39003682 PMC11560999

[17] Chen X Zhang T Hu X Wen Z Lu W Jiang W. High-intensity interval training programs versus moderate-intensity continuous training for individuals with heart failure: a systematic review and Meta-analysis. Arch Phys Med Rehabil. (2025) 106:98–112. doi: 10.1016/j.apmr.2024.05.028, 38862032

[18] Guo Z Li M Cai J Gong W Liu Y Liu Z. Effect of high-intensity interval training vs. moderate-intensity continuous training on fat loss and cardiorespiratory fitness in the young and middle-aged a systematic review and meta-analysis. Int J Environ Res Public Health. (2023) 20:741. doi: 10.3390/ijerph20064741, 36981649 PMC10048683

[19] Liang W Liu C Yan X Hou Y Yang G Dai J . The impact of sprint interval training versus moderate intensity continuous training on blood pressure and cardiorespiratory health in adults: a systematic review and meta-analysis. PeerJ. (2024) 12:e17064. doi: 10.7717/peerj.17064, 38495758 PMC10944631

[20] Neuendorf T Haase R Schroeder S Schumann M Nitzsche N. Effects of high-intensity interval training on functional performance and maximal oxygen uptake in comparison with moderate intensity continuous training in cancer patients: a systematic review and meta-analysis. Support Care Cancer. (2023) 31:643. doi: 10.1007/s00520-023-08103-9, 37851104 PMC10584719

[21] Ramos JS Dalleck LC Tjonna AE Beetham KS Coombes JS. The impact of high-intensity interval training versus moderate-intensity continuous training on vascular function: a systematic review and meta-analysis. Sports Med. (2015) 45:679–92. doi: 10.1007/s40279-015-0321-z, 25771785

[22] Sabag A Barr L Armour M Armstrong A Baker CJ Twigg SM . The effect of high-intensity interval training vs moderate-intensity continuous training on liver fat: a systematic review and Meta-analysis. J Clin Endocrinol Metab. (2022) 107:862–81. doi: 10.1210/clinem/dgab795, 34724062

[23] Yu H Zhao X Wu X Yang J Wang J Hou L. High-intensity interval training versus moderate-intensity continuous training on patient quality of life in cardiovascular disease: a systematic review and meta-analysis. Sci Rep. (2023) 13:13915. doi: 10.1038/s41598-023-40589-5, 37626066 PMC10457360

[24] Anjos JM Neto MG Dos Santos FS Almeida KO Bocchi EA Lima Bitar YS . The impact of high-intensity interval training on functioning and health-related quality of life in post-stroke patients: a systematic review with meta-analysis. Clin Rehabil. (2022) 36:726–39. doi: 10.1177/02692155221087082, 35290104

[25] Moncion K Rodrigues L Wiley E Noguchi KS Negm A Richardson J . Aerobic exercise interventions for promoting cardiovascular health and mobility after stroke: a systematic review with Bayesian network meta-analysis. Br J Sports Med. (2024) 58:392–400. doi: 10.1136/bjsports-2023-107956, 38413134

[26] Saipan P Kooncumchoo P Yuenyongchaiwat K Rungroungdouyboon B Muanjai P Sukkho O . Locomotor recovery following 8 weeks of I-walk gait training in subacute stroke. J Exerc Physiol. (2024) 27:11.

[27] Stoller O De Bruin ED Knols RH Hunt KJ. Effects of cardiovascular exercise early after stroke: systematic review and meta-analysis. BMC Neurol. (2012) 12:45. doi: 10.1186/1471-2377-12-45, 22727172 PMC3495034

[28] Sterne JaC Savović J Page MJ Elbers RG Blencowe NS Boutron I . RoB 2: a revised tool for assessing risk of bias in randomised trials. BMJ. (2019) 366:l4898. doi: 10.1136/bmj.l4898, 31462531

[29] De Morton NA. The PEDro scale is a valid measure of the methodological quality of clinical trials: a demographic study. Aust J Physiother. (2009) 55:129–33. doi: 10.1016/s0004-9514(09)70043-1, 19463084

[30] Smart NA Waldron M Ismail H Giallauria F Vigorito C Cornelissen V . Validation of a new tool for the assessment of study quality and reporting in exercise training studies: TESTEX. Int J Evid Based Healthc. (2015) 13:9–18. doi: 10.1097/XEB.0000000000000020, 25734864

[31] Hozo SP Djulbegovic B Hozo I. Estimating the mean and variance from the median, range, and the size of a sample. BMC Med Res Methodol. (2005) 5:13. doi: 10.1186/1471-2288-5-13, 15840177 PMC1097734

[32] Dersimonian R Laird N. Meta-analysis in clinical trials revisited. Contemp Clin Trials. (2015) 45:139–45. doi: 10.1016/j.cct.2015.09.002, 26343745 PMC4639420

[33] Balshem H Helfand M Schünemann HJ Oxman AD Kunz R Brozek J . GRADE guidelines: 3. Rating the quality of evidence. J Clin Epidemiol. (2011) 64:401–6. doi: 10.1016/j.jclinepi.2010.07.015, 21208779

[34] Boyne P Billinger SA Reisman DS Awosika OO Buckley S Burson J . Optimal intensity and duration of walking rehabilitation in patients with chronic stroke: a randomized clinical trial. JAMA Neurol. (2023) 80:342–51. doi: 10.1001/jamaneurol.2023.0033, 36822187 PMC9951105

[35] Boyne P Dunning K Carl D Gerson M Khoury J Rockwell B . High-intensity interval training and moderate-intensity continuous training in ambulatory chronic stroke: feasibility study. Phys Ther. (2016) 96:1533–44. doi: 10.2522/ptj.20150277, 27103222 PMC5046191

[36] Hsu CC Fu TC Huang SC Chen CP Wang JS. Increased serum brain-derived neurotrophic factor with high-intensity interval training in stroke patients: a randomized controlled trial. Ann Phys Rehabil Med. (2021) 64:101385. doi: 10.1016/j.rehab.2020.03.010, 32344098

[37] Lapointe T Houle J Sia YT Payette M Trudeau F. Addition of high-intensity interval training to a moderate intensity continuous training cardiovascular rehabilitation program after ischemic cerebrovascular disease: a randomized controlled trial. Front Neurol. (2022) 13:963950. doi: 10.3389/fneur.2022.963950, 36686521 PMC9846748

[38] Mahrukh M Riaz B Sharif Z Mahmood U Azfar H Ilyas MJ . Efficacy of high-intensity interval training versus moderate-intensity continuous training in chronic stroke rehabilitation. J Health Rehab Res. (2023) 3:187–93.

[39] Marzolini S Robertson AD Macintosh BJ Corbett D Anderson ND Brooks D . Effect of high-intensity interval training and moderate-intensity continuous training in people with poststroke gait dysfunction: a randomized clinical trial. J Am Heart Assoc. (2023) 12:e031532. doi: 10.1161/jaha.123.031532, 37947080 PMC10727274

[40] Moncion K Rodrigues L De Las Heras B Noguchi KS Wiley E Eng JJ . Cardiorespiratory fitness benefits of high-intensity interval training after stroke: a randomized controlled trial. Stroke. (2024) 55:2202–11. doi: 10.1161/STROKEAHA.124.046564, 39113181

[41] Rodrigues L Moncion K Angelopoulos SA Heras BL Sweet S Eng JJ . Psychosocial responses to a cardiovascular exercise randomized controlled trial: does intensity matter for individuals post-stroke? Arch Phys Med Rehabil. (2025) 106:468. doi: 10.1016/j.apmr.2025.01.468, 39894292

[42] Soh SH Joo MC Yun NR Kim MS. Randomized controlled trial of the lateral push-off skater exercise for high-intensity interval training vs conventional treadmill training. Arch Phys Med Rehabil. (2020) 101:187–95. doi: 10.1016/j.apmr.2019.08.480, 31562872

[43] Coffey VG Hawley JA. The molecular bases of training adaptation. Sports Med. (2007) 37:737–63. doi: 10.2165/00007256-200737090-00001, 17722947

[44] MacInnis MJ Gibala MJ. Physiological adaptations to interval training and the role of exercise intensity. J Physiol. (2017) 595:2915–30. doi: 10.1113/JP273196, 27748956 PMC5407969

[45] Metcalfe RS Koumanov F Ruffino JS Stokes KA Holman GD Thompson D . Physiological and molecular responses to an acute bout of reduced-exertion high-intensity interval training (REHIT). Eur J Appl Physiol. (2015) 115:2321–34. doi: 10.1007/s00421-015-3217-6, 26156806

[46] Fu TC Wang CH Lin PS Hsu CC Cherng WJ Huang SC . Aerobic interval training improves oxygen uptake efficiency by enhancing cerebral and muscular hemodynamics in patients with heart failure. Int J Cardiol. (2013) 167:41–50. doi: 10.1016/j.ijcard.2011.11.086, 22197120

[47] Li X He Y Wang D Rezaei MJ. Stroke rehabilitation: from diagnosis to therapy. Front Neurol. (2024) 15:1402729. doi: 10.3389/fneur.2024.1402729, 39193145 PMC11347453

[48] Li H Shih MC Song CJ Tu YK. Bias propagation in network meta-analysis models. Res Synth Methods. (2023) 14:247–65. doi: 10.1002/jrsm.1614, 36507611

[49] Poole DC Jones AM. Measurement of the maximum oxygen uptake V̇o2max: V̇o2peak is no longer acceptable. J Appl Physiol (1985). (2017) 122:997–1002. doi: 10.1152/japplphysiol.01063.2016, 28153947

[50] Liberati A Altman DG Tetzlaff J Mulrow C Gøtzsche PC Ioannidis JP . The PRISMA statement for reporting systematic reviews and meta-analyses of studies that evaluate healthcare interventions: explanation and elaboration. BMJ. (2009) 339:b2700. doi: 10.1016/j.jclinepi.2009.06.006, 19622552 PMC2714672

[51] Kodama S Saito K Tanaka S Maki M Yachi Y Asumi M . Cardiorespiratory fitness as a quantitative predictor of all-cause mortality and cardiovascular events in healthy men and women: a meta-analysis. JAMA. (2009) 301:2024–35. doi: 10.1001/jama.2009.681, 19454641

[52] Mitchell BL Lock MJ Davison K Parfitt G Buckley JP Eston RG . What is the effect of aerobic exercise intensity on cardiorespiratory fitness in those undergoing cardiac rehabilitation? A systematic review with meta-analysis.. Br J Sports Med.. (2019) 53:1341–51. doi: 10.1136/bjsports-2018-099153, 30121584

