# Supplementary material for: Effects of high-intensity interval training versus moderate-intensity continuous training on cardiorespiratory function in patients after stroke: a systematic review and meta-analysis of randomized trials
Source: Front Neurol. 2026 Feb 12;17:1727980. doi: 10.3389/fneur.2026.1727980 (PMC12937137; doi:10.3389/fneur.2026.1727980)
Supplement: Supplementary file 1 [file Supplementary_file_1.docx]

Supplementary Material

Supplementary Data 1 Search Strategy

1. **Pubmed:**

#1. "High-Intensity Interval Training"[Mesh] OR HIIT

#2. “Moderate-Intensity Continuous Training”[Mesh] OR MICT

#3. “Aerobic Training”

#4. "Stroke"[Mesh] OR cerebrovascular accident OR cerebrovascular disorder OR cerebral infarction OR brain infarction OR intracranial arteriosclerosis OR intracranial thrombosis OR intracranial embolism OR CVA

(#1. OR #2. OR #3.) AND #4.: **8171 Results**

1. **EMBASE:**

- ('cerebrovascular accident'/exp OR 'cva' OR 'accident, cerebrovascular' OR 'acute cerebrovascular lesion' OR 'acute focal cerebral vasculopathy' OR 'acute stroke' OR 'apoplectic stroke' OR 'apoplexia' OR 'apoplexy' OR 'blood flow disturbance, brain' OR 'brain accident' OR 'brain attack' OR 'brain blood flow disturbance' OR 'brain insult' OR 'brain insultus' OR 'brain vascular accident' OR 'cerebral apoplexia' OR 'cerebral insult' OR 'cerebral stroke' OR 'cerebral vascular accident' OR 'cerebral vascular insufficiency' OR 'cerebro vascular accident' OR 'cerebrovascular accident' OR 'cerebrovascular arrest' OR 'cerebrovascular failure' OR 'cerebrovascular injury' OR 'cerebrovascular insufficiency' OR 'cerebrovascular insult' OR 'cerebrum vascular accident' OR 'cryptogenic stroke' OR 'insultus cerebralis' OR 'ischaemic seizure' OR 'ischemic seizure' OR 'stroke' OR 'thrombotic stroke') AND (('high intensity interval training'/exp OR 'hiie (exercise)' OR 'hiit' OR 'high intensity interval training' OR 'high-intensity intermittent exercise' OR 'high-intensity intermittent training' OR 'high-intensity interval exercise' OR 'high-intensity interval training' OR 'intermittent high-intensity training' OR 'interval high-intensity training') OR ('moderate intensity continuous training'/exp OR 'mict (training)' OR 'continuous medium intensity training' OR 'continuous moderate intensity exercise' OR 'continuous moderate intensity training' OR 'medium intensity continuous training' OR 'moderate intensity continuous exercise' OR 'moderate intensity continuous training' OR 'aerobic exercise'/exp OR 'aerobic dance' OR 'aerobic dancing' OR 'aerobic exercise' OR 'aerobics' OR 'aerobics exercise' OR 'dancing, aerobic' OR 'exercise, aerobic' OR 'low impact aerobic exercise' OR 'low impact aerobics' OR 'step aerobics')): **1,934 Results**

1. **Cochrane Library**

- ((high-intensity interval training OR HIIT) OR (moderate-intensity continuous training OR MICT) OR aerobic training) AND (stroke OR cerebrovascular accident OR cerebrovascular disorder OR cerebral infarction OR brain infarction OR intracranial arteriosclerosis OR intracranial thrombosis OR intracranial embolism OR CVA): :ti,ab,kw **870 Results**
- **Supplementary Data** **2** Outcome protocols for assessing peak VO_2_

1. **Boyne et al., 2016 & Boyne et al., 2023:**

Aerobic capacity was measured using a maximal-effort GXT by the maximum 30-second average oxygen consumption (V̇O_2_) recording (V̇O_2peak_) and by V̇O_2_ at the ventilatory aerobic threshold (V̇O_2VAT_). Peak V̇O_2_ is the most common measure of aerobic capacity across populations and was our a priori primary outcome measure. Oxygen consumption at the ventilatory aerobic threshold represents the transition point from aerobic to anaerobic metabolism and is the upper intensity limit of prolonged activity. It was determined according to published guidelines, using a combination of the V-slope and ventilatory equivalents methods. The GXT followed the protocol of Macko et al, which has shown reliability among people with stroke. Briefly, speed was held constant at approximately 85% of the fastest comfortable speed from a steep ramp test (see fastest treadmill speed below), and incline was increased by 2% to 4% every 2 minutes. Test termination criteria included volitional fatigue, severe gait instability, or a cardiovascular safety limit.10 Respiratory gases were measured with the TrueOne 2400 (Parvo Medics, Sandy, Utah), using a facemask interface. Aerobic capacity measures obtained for each participant included V̇O_2peak_, OUES, and V̇O_2-VT_. V̇O_2peak_ was calculated as the highest 20 s average oxygen consumption rate recorded during the GXT. OUES was calculated using the equation: V̇O_2_ (mL/ min) = OUES × log10 VE (L/min) + intercept; where OUES is the slope of the regression line of V̇O_2_ vs. log transformed expiratory flow volume. V̇O_2-VT_ was determined using a combination of the V-slope and ventilatory equivalents methods.

1. **Hsu et al., 2021:**

Participants underwent an incremental exercise test on a bicycle ergometer (Ergoselect 150P, ergoline GmbH, Bitz, Germany) at an increased work-rate of 10 W/min with continuous monitoring of heart rate (HR), brachial blood pressure, and arterial oxygen saturation, until the stop conditions described previously. This examination was performed 2 days before exercise training and 2 days after completing exercise training. Oxygen consumption (VO_2_) was measured by a cardiopulmonary measurement device (MasterScreen CPX, CareFusion Corp., Hoechberg, Germany). VO_2peak_ was defined by: (1) level of VO2 increased < 2 mL/kg/min over least 2 min; (2) HR > 85% of predicted maximal value; (3) respiratory exchange ratio > 1.15 or; (4) some other symptom/sign limitations, as per the ACSM guideline for exercise testing methods.

1. **Lapointe et al., 2023:**

The GXT protocol was performed on a semi-recumbent ergocycle with 12-lead ECG monitoring (MAC 5500HD, GE Healthcare, USA). The cadence was maintained at 60 rpm; power started between 0 and 60 watts depending on the estimated participant’s capacity and increased progressively by 10 watts per minute. Blood pressure response was assessed manually with a sphygmomanometer (Hillrom, Welch Allyn Tycos, USA) every 3 min. Systolic blood pressure > 250 mm Hg or diastolic blood pressure > 115 mm Hg were considered an absolute indication for terminating the GXT. According to ACSM guidelines, test termination criteria also included volitional fatigue, significant arrhythmia, evidence of ischemia, angina-like symptoms, a drop in systolic blood pressure of ≥10 mm Hg despite an increase in work rate or below the value obtained on the ergocycle prior to testing, shortness of breath, wheezing or leg cramps, signs of poor perfusion, failure of heart rate to increase with increased intensity or the participant’s request to stop. The majority of GXT (95%) were stopped because of volitional fatigue, and other reasons were a patient’s request to stop because of pain in the leg (4%) and a decrease of systolic blood pressure below the prior test value (1%). PPO was established as the highest power maintained during 60 s during the final stage of the GXT and was used to determine exercise intensity during the exercise program. This value was used to estimate VO_2peak_ using the ACSM equation. The GXT protocol was also assessed at 3 months (T3) in the two exercise groups to adjust exercise intensity.

1. **Marhkurh et al., 2023:**

The tools employed for data collection included a cardiopulmonary exercise test to measure VO_2max_, based on the following article:

Mazaheri, R., Schmied, C., Niederseer, D., and Guazzi, M. (2021). Cardiopulmonary Exercise Test Parameters in Athletic Population: A Review. *J Clin Med* 10, 5073.

1. **Marzolini et al., 2023;**

V̇O_2_ was measured using an ambulatory oxygen monitor (CORTEX Biophysik MetaMax 3X portable gas analyzer, Germany).3 This technology allows for reliable and accurate measures of oxygen uptake in stroke participants.

1. **Monicon et al., 2024:**

Each participant completed a symptom-limited CPET on a recumbent stepper. Participants wore a face mask connected to a calibrated metabolic system (Quark CPET, COSMED SrI, Rome, Italy). Participants were instructed to maintain a stepping cadence of at least 80 steps per minute throughout the CPET. The CPET was terminated if participants reached volitional fatigue, achieved any of the absolute termination criteria for exercise testing, or were unable to maintain step cadence after 2 warnings. Blood pressure (Dinamap V100; General Electric Healthcare, Chicago, IL), HR (Polar H10 Heart Rate Sensor; Polar Electro, Kempele, Finland), and RPE were monitored throughout the CPET.

1. **Soh et al., 2020:**

The CRF of the participants was assessed with the cardiorespiratory exercise test using the modified Bruce protocol. The indicators for CRF were investigated by dividing them into aerobic capacity, cardiovascular response, and ventilator response. Aerobic capacity was determined by peak oxygen consumption (VO_2peak_), the criterion standard parameter for assessing one’s functional capacity.

# Supplementary Table 1 Methodological quality assessment of the selected randomized controlled trials with PEDro scale

| Study  (Year) | Eligibility criteria | Random  allocation | Concealed allocation | Baseline comparability | Blinded participants | Blinded therapists | Blinded assessors | Adequate follow-up | Intention-to-treat analysis | Between-group comparisons | Point estimated and variability | Total PEDro score |
| --- | --- | --- | --- | --- | --- | --- | --- | --- | --- | --- | --- | --- |
| Boyne  (2016) | 1 | 1 | 1 | 1 | 0 | 0 | 1 | 1 | 1 | 1 | 1 | 8 |
| Boyne  (2023) | 1 | 1 | 1 | 1 | 0 | 0 | 1 | 1 | 1 | 1 | 1 | 8 |
| Hsu (2021) | 1 | 1 | 1 | 1 | 0 | 0 | 1 | 0 | 0 | 1 | 1 | 6 |
| Lapointe (2023) | 1 | 1 | 0 | 1 | 0 | 0 | 0 | 0 | 0 | 1 | 1 | 4 |
| Mahrukh (2023) | 1 | 1 | 0 | 0 | 0 | 0 | 0 | 0 | 1 | 1 | 1 | 4 |
| Marzolini (2023) | 1 | 1 | 1 | 1 | 0 | 0 | 1 | 1 | 1 | 1 | 1 | 8 |
| Moncion (2024)  Rodrigues. (2025) | 1 | 1 | 1 | 1 | 0 | 0 | 0 | 0 | 1 | 1 | 1 | 6 |
| Soh  (2020) | 1 | 1 | 1 | 1 | 0 | 0 | 0 | 0 | 1 | 1 | 0 | 5 |

# Supplementary Table 2 Methodological quality assessment of the selected randomized controlled trials with TESTEX scale

| Study  (Year) | Eligibility criteria specified | Randomly allocated participants | Allocation concealed | Groups similar at Baseline | Assessors blinded | Outcome measures assess >85% of participants ^a^ | Intention-to-treat analysis | Reporting of between group statistical comparison | Point measures and measures of variability reported | Activity monitoring in control group | Relative exercise intensity review | Exercise volume and energy expended | Total TESTEX score |
| --- | --- | --- | --- | --- | --- | --- | --- | --- | --- | --- | --- | --- | --- |
| Boyne  (2016) | 1 | 1 | 1 | 1 | 1 | 3 | 1 | 2 | 1 | 1 | 1 | 1 | 15 |
| Boyne  (2023) | 1 | 1 | 1 | 1 | 1 | 2 | 1 | 2 | 1 | 1 | 1 | 1 | 14 |
| Hsu (2021) | 1 | 1 | 1 | 1 | 1 | 1 | 0 | 2 | 1 | 1 | 1 | 1 | 12 |
| Lapointe (2023) | 1 | 1 | 0 | 1 | 0 | 2 | 0 | 2 | 1 | 1 | 1 | 1 | 11 |
| Mahrukh (2023) | 1 | 1 | 0 | 0 | 0 | 1 | 1 | 2 | 1 | 1 | 1 | 1 | 10 |
| Marzolini (2023) | 1 | 1 | 1 | 1 | 1 | 3 | 1 | 2 | 1 | 1 | 1 | 1 | 15 |
| Moncion (2024)  Rodrigues. (2025) | 1 | 1 | 1 | 1 | 0 | 2 | 1 | 2 | 1 | 1 | 1 | 1 | 13 |
| Soh  (2020) | 1 | 1 | 1 | 1 | 0 | 2 | 1 | 2 | 1 | 1 | 1 | 1 | 13 |

a: 1 point if completion rate is >85%; 1 point if adverse events are reported; 1 point if exercise attendance is reported.

b: 1 point if between-group statistical comparisons are reported for the primary outcome measure of interest; 1 point if between-group statistical comparisons are reported for at least one secondary outcome measure

# Supplementary Table 3 GRADE Quality of Evidence for HIIT compared with MICT

| **Certainty assessment** | | | | | | | | **№ of patients** | | **Effect** | **Certainty** |
| --- | --- | --- | --- | --- | --- | --- | --- | --- | --- | --- | --- |
| **№ of studies** | | | **Risk of bias** | **Inconsistency** | **Indirectness** | **Imprecision** | **Publication bias** | **HIIT** | **MICT** | **Absolute (95% CI)** |  |
| **V̇O_2-peak_ (mL/kg/mi)** | | | | | | | | | | | |
| 8 | | | not serious | not serious | not serious | serious ^a^ | Not significant | 163 | 157 | MD **1.88** mL/kg/min  (1.20 to 2.55) | ⨁⨁⨁◯ Moderate |
| **VO_2-VT_ (mL/kg/mi)** | | | | | | | | | | | |
| 3 | | | not serious | very serious ^b^ | not serious | serious ^a^ | Not significant | 59 | 49 | MD **2.20** mL/kg/min  (0.46 to 3.95) | ⨁◯◯◯  Very low |
| **6-minute Walk Distance (m)** | | | | | | | | | | | |
| 5 | | | not serious | very serious ^b^ | not serious | serious ^a^ | Not significant | 134 | 126 | MD **17.63** m  (−1.44 to 36.70) | ⨁◯◯◯  Very low |
| **10-meter Gait Speed (m/s)** | | | | | | | | | | | |
| 4 | | | not serious | serious ^b^ | not serious | serious ^a^ | Not significant | 4 | 96 | MD **0.08** m/s  (−0.01 to 0.17) | ⨁⨁◯◯  Low |
| **Berg Balance Score (0-56 points)** | | | | | | | | | | | |
| 2 | | | not serious | very serious ^b^ | not serious | serious ^a^ | Not significant | 38 | 40 | MD **2.57** points  (−4.53 to 9.68) | ⨁◯◯◯  Very low |
| **Training Fidelity - Mean Heart Rate (%HRR)** | | | | | | | | | | | |
| 3 | | | not serious | not serious | not serious | serious ^a^ | Not significant | 57 | 57 | MD **19.36 higher**  (13.83 to 24.90) | ⨁⨁⨁◯ Moderate |
| **Training Fidelity - Peak Heart Rate** | | | | | | | | | |  |  |
| 3 | not serious | very serious ^b^ | not serious | serious ^a^ | Not significant | 64 | 65 | SMD **1.00 higher**  (0.40 to 1.59) | ⨁◯◯◯  Very low |  |  |
| **Resting Systolic Blood Pressure (mmHg)** | | | | | | | | | | |  |
| 4 | | not serious | very serious ^b^ | not serious | serious ^a^ | Not significant | 103 | 97 | MD **1.72 lower**  (5.92 lower to 2.48 higher) | ⨁◯◯◯  Very low |  |
| **Resting Diastolic Blood Pressure (mmHg)** | | | | | | | | | | |  |
| 4 | | not serious | very serious ^b^ | not serious | serious ^a^ | Not significant | 103 | 97 | MD **1.04 lower**  (3.78 lower to 1.69 higher) | ⨁◯◯◯  Very low |  |

^a^ The numbers of included studies and patients are small.

^b^ Unexplained heterogeneity was detected in the pooled result.

# Supplementary Table 4 PRISMA Checklist

| **Section and Topic** | **Item #** | **Checklist item** | **Location where item is reported** |
| --- | --- | --- | --- |
| **TITLE** | | |  |
| Title | 1 | Identify the report as a systematic review. | Title |
| **ABSTRACT** | | |  |
| Abstract | 2 | See the PRISMA 2020 for Abstracts checklist. | Abstract |
| **INTRODUCTION** | | |  |
| Rationale | 3 | Describe the rationale for the review in the context of existing knowledge. | Section 1 |
| Objectives | 4 | Provide an explicit statement of the objective(s) or question(s) the review addresses. | Section 1 |
| **METHODS** | | |  |
| Eligibility criteria | 5 | Specify the inclusion and exclusion criteria for the review and how studies were grouped for the syntheses. | Section 2.2 |
| Information sources | 6 | Specify all databases, registers, websites, organisations, reference lists and other sources searched or consulted to identify studies. Specify the date when each source was last searched or consulted. | Section 2.1 |
| Search strategy | 7 | Present the full search strategies for all databases, registers and websites, including any filters and limits used. | Section 2.1 |
| Selection process | 8 | Specify the methods used to decide whether a study met the inclusion criteria of the review, including how many reviewers screened each record and each report retrieved, whether they worked independently, and if applicable, details of automation tools used in the process. | Section 2.2 |
| Data collection process | 9 | Specify the methods used to collect data from reports, including how many reviewers collected data from each report, whether they worked independently, any processes for obtaining or confirming data from study investigators, and if applicable, details of automation tools used in the process. | Section 2.3 |
| Data items | 10a | List and define all outcomes for which data were sought. Specify whether all results that were compatible with each outcome domain in each study were sought (e.g. for all measures, time points, analyses), and if not, the methods used to decide which results to collect. | Section 2.3 |
|  | 10b | List and define all other variables for which data were sought (e.g. participant and intervention characteristics, funding sources). Describe any assumptions made about any missing or unclear information. | Section 2.3 |
| Study risk of bias assessment | 11 | Specify the methods used to assess risk of bias in the included studies, including details of the tool(s) used, how many reviewers assessed each study and whether they worked independently, and if applicable, details of automation tools used in the process. | Section 2.5 |
| Effect measures | 12 | Specify for each outcome the effect measure(s) (e.g. risk ratio, mean difference) used in the synthesis or presentation of results. | Section 2.4 |
| Synthesis methods | 13a | Describe the processes used to decide which studies were eligible for each synthesis (e.g. tabulating the study intervention characteristics and comparing against the planned groups for each synthesis (item #5)). | Section 2.6 |
|  | 13b | Describe any methods required to prepare the data for presentation or synthesis, such as handling of missing summary statistics, or data conversions. | Section 2.6 |
|  | 13c | Describe any methods used to tabulate or visually display results of individual studies and syntheses. | Section 2.6 |
|  | 13d | Describe any methods used to synthesize results and provide a rationale for the choice(s). If meta-analysis was performed, describe the model(s), method(s) to identify the presence and extent of statistical heterogeneity, and software package(s) used. | Section 2.6 |
|  | 13e | Describe any methods used to explore possible causes of heterogeneity among study results (e.g. subgroup analysis, meta-regression). | Section 2.6 |
|  | 13f | Describe any sensitivity analyses conducted to assess robustness of the synthesized results. | Section 2.6 |
| Reporting bias assessment | 14 | Describe any methods used to assess risk of bias due to missing results in a synthesis (arising from reporting biases). | Section 2.6 |
| Certainty assessment | 15 | Describe any methods used to assess certainty (or confidence) in the body of evidence for an outcome. | Section 2.7 |
| **RESULTS** | | |  |
| Study selection | 16a | Describe the results of the search and selection process, from the number of records identified in the search to the number of studies included in the review, ideally using a flow diagram. | Section 3.1 |
|  | 16b | Cite studies that might appear to meet the inclusion criteria, but which were excluded, and explain why they were excluded. | Section 3.1 |
| Study characteristics | 17 | Cite each included study and present its characteristics. | Section 3.1 |
| Risk of bias in studies | 18 | Present assessments of risk of bias for each included study. | Section 3.2 |
| Results of individual studies | 19 | For all outcomes, present, for each study: (a) summary statistics for each group (where appropriate) and (b) an effect estimate and its precision (e.g. confidence/credible interval), ideally using structured tables or plots. | Sec 3.3-3.7 |
| Results of syntheses | 20a | For each synthesis, briefly summarise the characteristics and risk of bias among contributing studies. | Sec 3.3-3.7 |
|  | 20b | Present results of all statistical syntheses conducted. If meta-analysis was done, present for each the summary estimate and its precision (e.g. confidence/credible interval) and measures of statistical heterogeneity. If comparing groups, describe the direction of the effect. | Sec 3.3-3.7 |
|  | 20c | Present results of all investigations of possible causes of heterogeneity among study results. | Sec 3.3-3.7 |
|  | 20d | Present results of all sensitivity analyses conducted to assess the robustness of the synthesized results. | Sec 3.3-3.7 |
| Reporting biases | 21 | Present assessments of risk of bias due to missing results (arising from reporting biases) for each synthesis assessed. | Sec 3.3-3.7 |
| Certainty of evidence | 22 | Present assessments of certainty (or confidence) in the body of evidence for each outcome assessed. | Section 3.8 |
| **DISCUSSION** | | |  |
| Discussion | 23a | Provide a general interpretation of the results in the context of other evidence. | Section 4 |
|  | 23b | Discuss any limitations of the evidence included in the review. | Section 5 |
|  | 23c | Discuss any limitations of the review processes used. | Section 5 |
|  | 23d | Discuss implications of the results for practice, policy, and future research. | Section 6 |
| **OTHER INFORMATION** | | |  |
| Registration and protocol | 24a | Provide registration information for the review, including register name and registration number, or state that the review was not registered. | Abstract |
|  | 24b | Indicate where the review protocol can be accessed, or state that a protocol was not prepared. | Abstract |
|  | 24c | Describe and explain any amendments to information provided at registration or in the protocol. | Abstract |
| Support | 25 | Describe sources of financial or non-financial support for the review, and the role of the funders or sponsors in the review. | Funding |
| Competing interests | 26 | Declare any competing interests of review authors. | Conflict of interest |
| Availability of data, code and other materials | 27 | Report which of the following are publicly available and where they can be found: template data collection forms; data extracted from included studies; data used for all analyses; analytic code; any other materials used in the review. | Data availability statement |

*From:*  Page MJ, McKenzie JE, Bossuyt PM, Boutron I, Hoffmann TC, Mulrow CD, et al. The PRISMA 2020 statement: an updated guideline for reporting systematic reviews. BMJ 2021;372:n71. doi: 10.1136/bmj.n71

For more information, visit: <http://www.prisma-statement.org/>


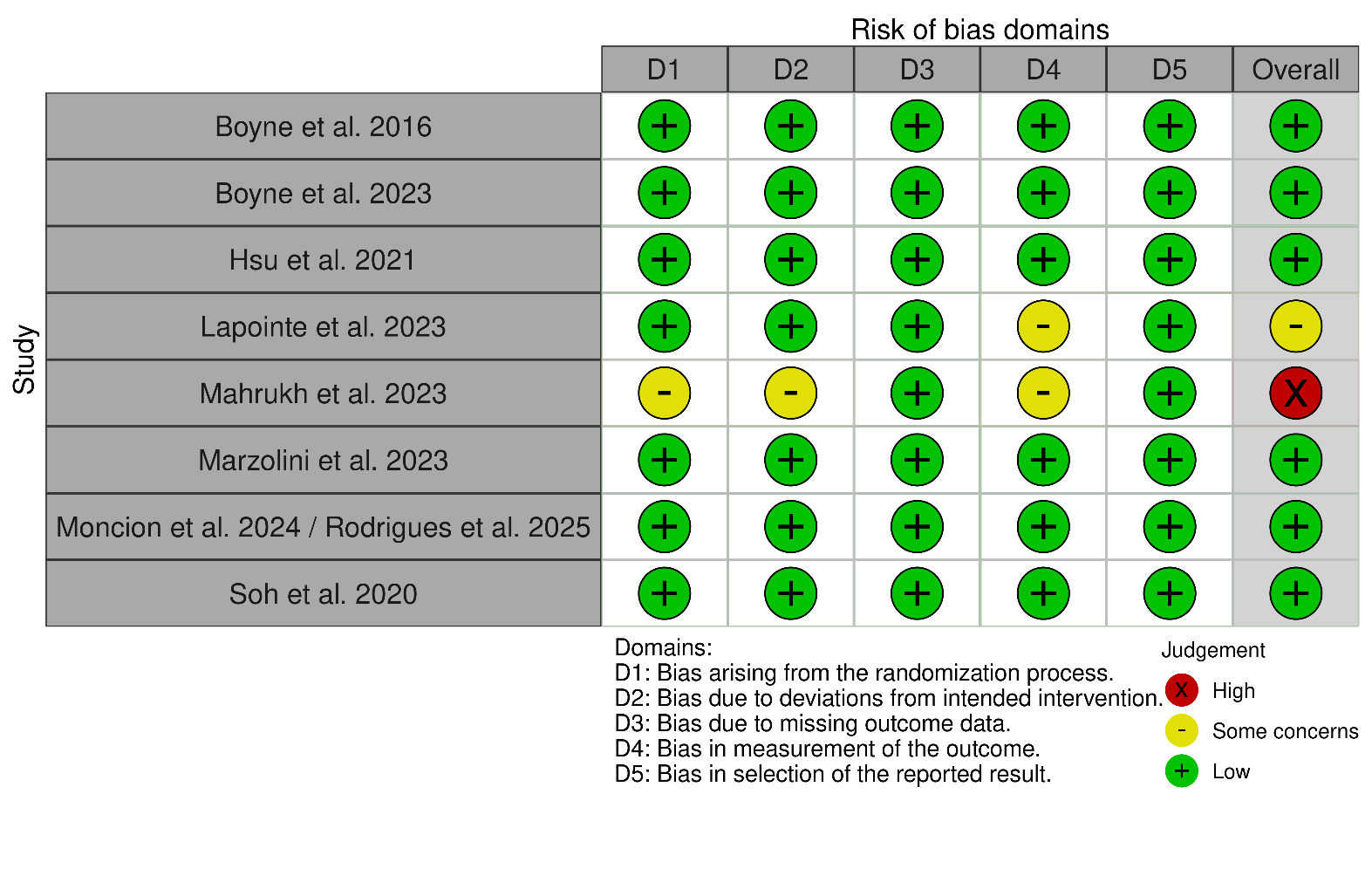


**Supplementary Figure 1** Methodological quality assessment of the selected randomized controlled trials (RoB 2.0)


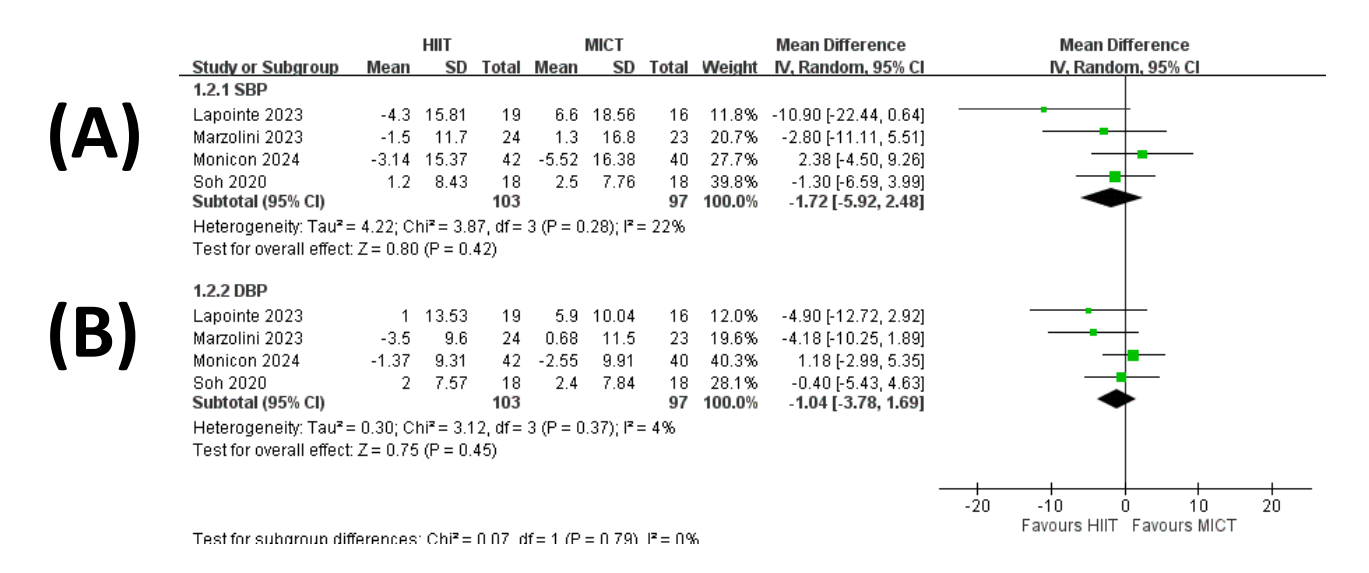


**Supplementary Figure 2** Forest Plot Presenting a Comparison of Changes in Resting Blood Pressure: (A) systolic blood pressure and (B) diastolic blood pressure. (mmHg)
